# [(1,2,5,6-η)-Cyclo­octa-1,5-diene](4-isopropyl-1-methyl-1,2,4-triazol-5-yl­idene)(tri­phenyl­phos­phane)iridium(I) tetra­fluorido­borate di­chloro­methane 0.8-solvate

**DOI:** 10.1107/S2414314623000640

**Published:** 2023-01-31

**Authors:** Troy E. Smith, Andrei V. Astashkin, Daniel R. Albert, Edward Rajaseelan

**Affiliations:** a Department of Chemistry and Biochemistry, The University of Arizona, Tuscon, AZ, 85716, USA; b Department of Chemistry, Millersville University, Millersville, PA 17551, USA; Vienna University of Technology, Austria

**Keywords:** crystal structure, iridium, N-heterocyclic carbenes

## Abstract

A new triazole-based N-heterocyclic carbene iridium(I) cationic complex with a tetra­fluorido­borate counter-anion, [Ir(C_8_H_12_)(C_18_H_15_P)(C_6_H_11_N_3_)]BF_4_·0.8CH_2_Cl_2_, has been synthesized and structurally characterized. The central Ir^I^ atom of the cationic complex has a distorted square-planar coordination environment, formed by a bidentate cyclo­octa-1,5-diene (COD) ligand, an N-heterocyclic carbene, and a tri­phenyl­phosphane ligand. The crystal structure comprises C—H⋯π(ring) inter­actions that orient the phenyl rings; non-classical hydrogen-bonding inter­actions between the cationic complex and the tetra­fluorido­borate anion are also present. The complex crystallizes in a triclinic unit cell with two structural units and an incorporation of di­chloro­methane solvate mol­ecules with an occupancy of 0.8.

## Abstract 2

The central Ir^I^ atom of the cationic complex of the title compound, [Ir(C_8_H_12_)(C_18_H_15_P)(C_6_H_11_N_3_)][BF_4_] ·0.8CH_2_Cl_2_, exhibits a distorted square-planar coordination environment.

## Graphical abstract

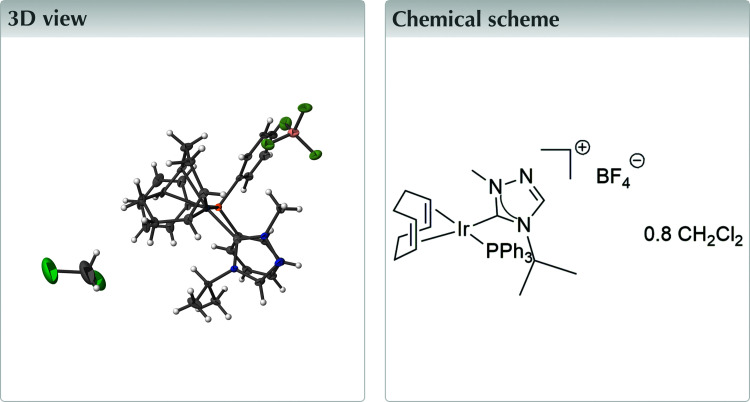

## Structure description

N-heterocyclic carbenes (NHC) have emerged as excellent ligands in transition-metal chemistry and in homogeneous catalysis (Cazin, 2013 ▸; de Frémont *et al.*, 2009 ▸; Diez-Gonzáles *et al.*, 2009 ▸; Rovis & Nolan, 2013 ▸; Ruff *et al.*, 2016 ▸; Zuo *et al.*, 2014 ▸). They have also shown catalytic activity in the transfer hydrogenation of ketones and imines (Albrecht *et al.*, 2002 ▸; Gnanamgari *et al.*, 2007 ▸). The NHC ligands can be tuned sterically and electronically by having different substituents on the nitro­gen atoms (Gusev, 2009 ▸). Many imidazole- and triazole-based NHC rhodium and iridium complexes have been synthesized and structurally characterized in the past (Herrmann *et al.*, 2006 ▸; Wang & Lin, 1998 ▸; Chianese *et al.*, 2004 ▸). As part of our ongoing research, we continue to synthesize new imidazole- and triazole-based NHC complexes of rhodium and iridium in order to study the effect of different substituents on the NHC and other ligands coordinating to the metal in transfer hydrogenation reactions (Nichol *et al.*, 2009 ▸, 2010 ▸, 2011 ▸, 2012 ▸; Idrees *et al.*, 2017*a* ▸,*b* ▸; Rood *et al.*, 2021 ▸; Rushlow *et al.*, 2021 ▸, 2022 ▸; Newman *et al.*, 2021 ▸; Castaldi *et al.*, 2021 ▸).

**Figure 1:**
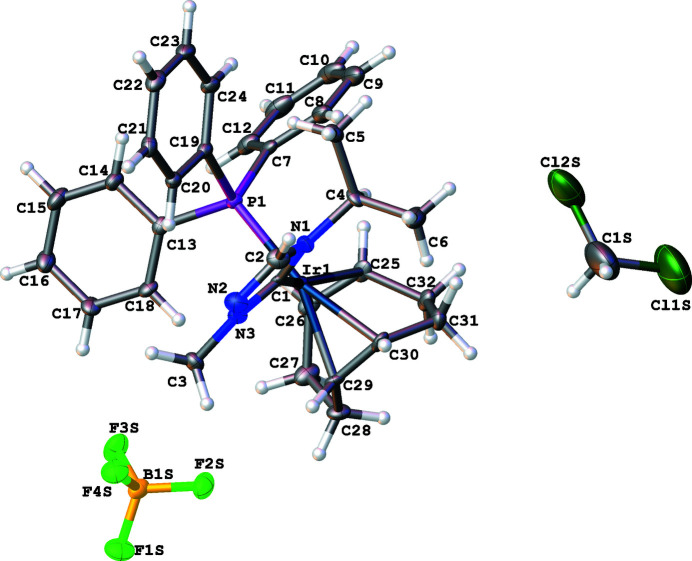
The mol­ecular entities in the crystal structure of the title compound **2**. Displacement ellipsoids are drawn at the 50% probability level.

The mol­ecular structure of the title complex **2**, shown in Fig. 1 ▸, is characterized as an Ir^I^ cationic complex with a tetra­fluorido­borate counter-ion, with partial incorporation of di­chloro­methane solvate mol­ecules (s.o.f. 0.8). The distorted square-planar environment around the Ir^I^ atom is defined by the bidentate cyclo­octa-1,5-diene (COD) ligand, the carbene C1 atom of the triazole NHC ligand, and the P atom of the tri­phenyl­phosphane ligand. The P1—Ir1—C1 bond angle is 93.88 (10)°. The N1—C1—N3 bond angle of the coordinating carbene atom significantly differs with a value of 103.3 (3)° from the expected *sp^2^* hybridization.

**Figure 2:**
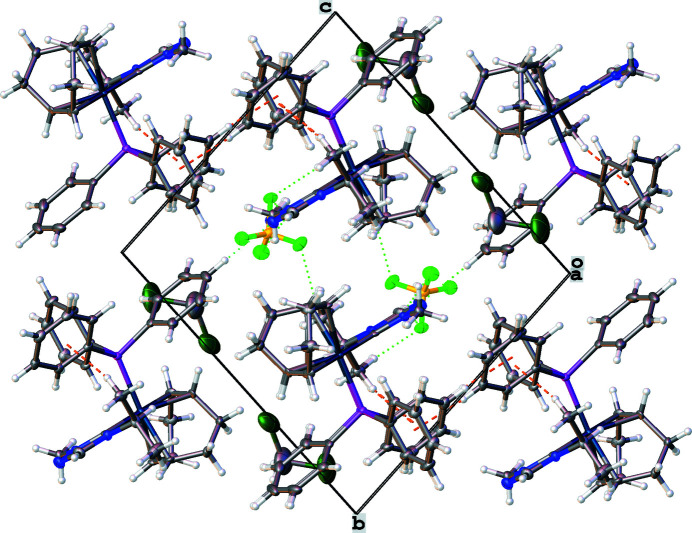
Crystal packing of the title compound **2** shown along the *a* axis. Non-classical hydrogen-bonding inter­actions are shown as dotted green lines. C—H⋯π(ring) inter­actions are shown as dashed orange lines between hydrogen atoms and phenyl ring centroids.

The crystal packing of the title compound is displayed in Fig. 2 ▸. There are several non-classical hydrogen-bonding inter­actions between the cation and anion that orient the [BF_4_]^−^ group. Additionally, there are non-classical inter­molecular hydrogen-bonding inter­actions between the hydrogen atom of a phenyl group (H10) and a nitro­gen atom of the NHC ligand (N2). Non-classical hydrogen bonding inter­actions are shown as dotted green lines in Fig. 2 ▸, and their numerical data summarized in Table 1 ▸. Notably absent are hydrogen-bonding inter­actions with the di­chloro­methane solvate. The lack of hydrogen-bonding inter­actions involving the solvate may contribute to its partial occupancy.

**Figure 3:**
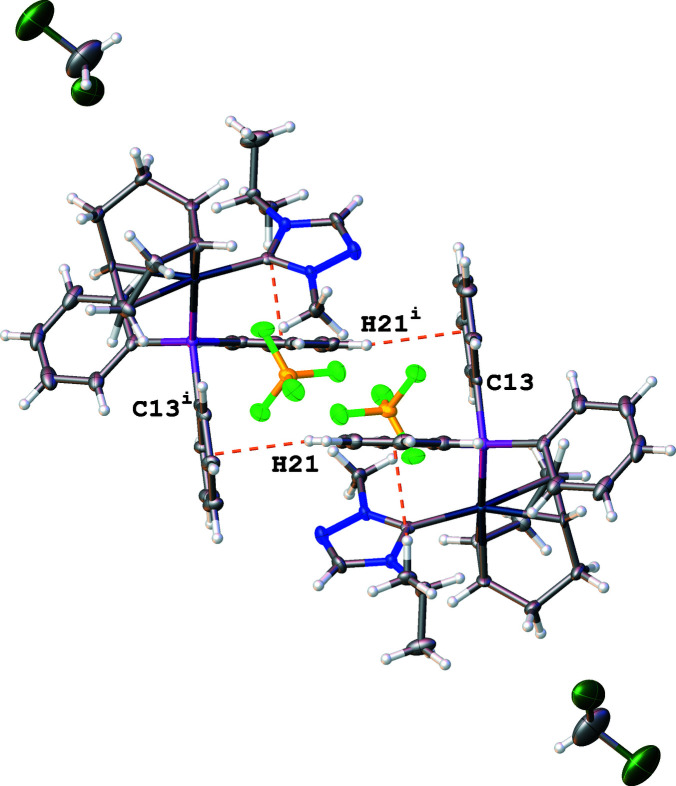
View of the title compound **2** showing perpendicular ring orientations arising from C—H⋯π(ring) inter­actions (shown as dashed orange lines). [Symmetry code: (i) −*x* + 1, −*y* + 1, −*z* + 2.]

Both inter­molecular and intra­molecular C—H⋯π(ring) inter­actions are observed and shown as dashed orange lines in Figs. 2 ▸ and 3 ▸. The intra­molecular C—H⋯π(ring) inter­action is between a hydrogen atom on the isopropyl wingtip of the NHC ligand (H5*A*) and a phenyl phosphane ring (C19–C24). This intra­molecular inter­action displays an H⋯centroid distance of 2.61 Å and a C—H⋯centroid angle of 168°. The inter­molecular C—H⋯π(ring) inter­action orients phenyl phosphane rings of adjacent moieties as it occurs between a hydrogen atom of a phenyl ring (H21) and an adjacent phenyl ring (C13–C18). The inter­molecular C—H⋯π(ring) inter­action has an H⋯centroid distance of 2.73 Å and a C—H⋯centroid angle of 157°. The C—H⋯π(ring) inter­actions orient phenyl rings on adjacent moieties (C13–C18 and C19–C24) into an approximately perpendicular arrangement, shown in Fig. 3 ▸, with a dihedral angle between the ring planes of 82.3 (2)°.

## Synthesis and crystallization

**Figure 4:**
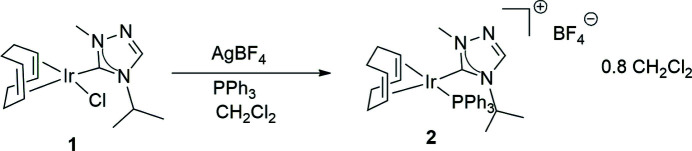
Reaction scheme for the synthesis of the title compound **2**.

**[(1,2,5,6-η)-Cyclo­octa-1,5-diene](4-isopropyl-1-methyl-1,2,4-triazol-5-yl­idene) chloro­iridium (1)** was synthesized by a previously published procedure (Rushlow *et al.*, 2022 ▸). The synthesis, shown schematically in Fig. 4 ▸, was performed under nitro­gen atmosphere using reagent grade materials purchased from Sigma–Aldrich and Strem, which were used as received without further purification. All NMR spectra were recorded at room temperature in CDCl_3_ on a 400 MHz (operating at 162 MHz for ^31^P) Varian spectrometer and referenced to the residual solvent peak of CDCl_3_ (δ in p.p.m.).

**[(1,2,5,6-η)-Cyclo­octa-1,5-diene](4-isopropyl-1-methyl-1,2,4-triazol-5-yl­idene)(tri­phenyl­phosphane)iridium(I) tetra­fluorido­borate (2):** Tri­phenyl­phosphane (0.064 g, 0.245 mmol) and AgBF_4_ (0.048 g 0.245 mmol) were added to an oven-dried flask containing complex (**1)** (0.113 g, 0.245 mmol) in 10 ml of CH_2_Cl_2_, and stirred under N_2_ in the dark for 90 min. The mixture was filtered through Celite and the solvent was removed under reduced pressure. The bright orange–red solid was washed with pentane and dried under vacuum yielding 0.165 g (86.9%) of the title compound **2**. ^1^H NMR: δ (p.p.m.) 8.18 (*s*, 1 H, N—C_3_H—N), 7.49–7.32 (*m*, 15 H, H_arom_), 5.36 (*m*, 1 H, CH(CH_3_)_2_), 4.38, 3.99 (*m*, 4 H, CH of COD), 4.05 (*s*, 3 H, CH_3_—N), 2.27–1.6 (*m*, CH_2_ of COD), 1.56 [*d*, 6 H, CH(CH_3_)_2_]. ^13^C NMR: δ 177.74 (Ir—C), 140.32 (N—CH—N), 132.46–128.38 (C_arom_), 87.82, 87.43, 85.34, 85.01 (CH of COD), 53.23 [CH(CH_3_)_2_], 41.31 (N—CH_3_), 33.41, 33.18, 31.45, 30.39 CH_2_ of COD, 24.37, 22.15 [CH(CH_3_)_2_]. ^31^P: δ 17.23.

The title compound **2** was crystallized by slow diffusion of pentane into a CH_2_Cl_2_ solution.

## Refinement

Crystal data, data collection, and structure refinement details are summarized in Table 2 ▸.

## Supplementary Material

Crystal structure: contains datablock(s) I. DOI: 10.1107/S2414314623000640/wm4181sup1.cif

Structure factors: contains datablock(s) I. DOI: 10.1107/S2414314623000640/wm4181Isup2.hkl

CCDC reference: 2237810

Additional supporting information:  crystallographic information; 3D view; checkCIF report

## Figures and Tables

**Table 1:** Hydrogen-bond geometry (Å, °)

*D*—H⋯*A*	*D*—H	H⋯*A*	*D*⋯*A*	*D*—H⋯*A*
C2—H2⋯F3*S* ^i^	0.95	2.60	3.471 (5)	153
C2—H2⋯F1*S* ^i^	0.95	2.30	3.154 (5)	149
C5—H5*C*⋯F3*S* ^i^	0.98	2.54	3.505 (5)	169
C6—H6*C*⋯F2*S* ^ii^	0.98	2.50	3.451 (5)	163
C10—H10⋯N2^iii^	0.95	2.42	3.364 (6)	172

Symmetry codes: (i) 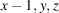 ; (ii) 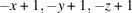 ; (iii) 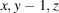 .

**Table 2:** Experimental details

Crystal data	
Chemical formula	[Ir(C_8_H_12_)(C_18_H_15_P)(C_6_H_11_N_3_)]BF_4_·0.8CH_2_Cl_2_
*M*_r_	842.57
Crystal system, space group	Triclinic, *P* 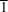
Temperature (K)	100
*a*, *b*, *c* (Å)	10.551 (3), 12.444 (4), 13.804 (5)
α, β, γ (°)	95.258 (10), 101.022 (9), 94.954 (10)
*V* (Å^3^)	1761.6 (10)
*Z*	2
Radiation type	Mo *K*α
μ (mm^−1^)	4.00
Crystal size (mm)	0.20 × 0.08 × 0.04

Data collection	
Diffractometer	Bruker APEXII CCD
Absorption correction	Multi-scan (*SADABS*; Krause *et al.*, 2015 ▸)
*T*_min_, *T*_max_	0.639, 0.745
No. of measured, independent and observed [*I* > 2σ(*I*)] reflections	31123, 7234, 6448
*R*_int_	0.042
(sin θ/λ)_max_ (Å^−1^)	0.626

Refinement	
*R*[*F* ^2^ > 2σ(*F* ^2^)], *wR*(*F* ^2^), *S*	0.028, 0.063, 1.04
No. of reflections	7234
No. of parameters	409
H-atom treatment	H-atom parameters constrained
Δρ_max_, Δρ_min_ (e Å^−3^)	1.41, −0.90

Computer programs: *APEX2* and *SAINT* (Bruker, 2013 ▸), *SHELXT* (Sheldrick, 2015*a* ▸), *SHELXL* (Sheldrick, 2015*b* ▸), *OLEX2* (Dolomanov *et al.*, 2009 ▸) and *publCIF* (Westrip, 2010 ▸).

## full crystallographic data

### Crystal data

**Table:**

[Ir(C_8_H_12_)(C_18_H_15_P)(C_6_H_11_N_3_)]BF_4_·0.8CH_2_Cl_2_	*Z* = 2
*M**_r_* = 842.57	*F*(000) = 835
Triclinic, *P*1	*D*_x_ = 1.589 Mg m^−^^3^
*a* = 10.551 (3) Å	Mo *K*α radiation, λ = 0.71073 Å
*b* = 12.444 (4) Å	Cell parameters from 9889 reflections
*c* = 13.804 (5) Å	θ = 2.4–25.8°
α = 95.258 (10)°	µ = 4.00 mm^−^^1^
β = 101.022 (9)°	*T* = 100 K
γ = 94.954 (10)°	Plate, clear light orange
*V* = 1761.6 (10) Å^3^	0.20 × 0.08 × 0.04 mm

### Data collection

**Table:**

Bruker APEXII CCD diffractometer	6448 reflections with *I* > 2σ(*I*)
Detector resolution: 8 pixels mm^-1^	*R*_int_ = 0.042
ω and φ scans	θ_max_ = 26.4°, θ_min_ = 1.7°
Absorption correction: multi-scan (*SADABS*; Krause *et al.*, 2015)	*h* = −13→13
*T*_min_ = 0.639, *T*_max_ = 0.745	*k* = −15→15
31123 measured reflections	*l* = −17→17
7234 independent reflections	

### Refinement

**Table:**

Refinement on *F*^2^	0 restraints
Least-squares matrix: full	Hydrogen site location: inferred from neighbouring sites
*R*[*F*^2^ > 2σ(*F*^2^)] = 0.028	H-atom parameters constrained
*wR*(*F*^2^) = 0.063	*w* = 1/[σ^2^(*F*_o_^2^) + (0.0283*P*)^2^ + 2.2709*P*] where *P* = (*F*_o_^2^ + 2*F*_c_^2^)/3
*S* = 1.04	(Δ/σ)_max_ = 0.002
7234 reflections	Δρ_max_ = 1.41 e Å^−^^3^
409 parameters	Δρ_min_ = −0.90 e Å^−^^3^

### Special details

**Table:**

Geometry. All esds (except the esd in the dihedral angle between two l.s. planes) are estimated using the full covariance matrix. The cell esds are taken into account individually in the estimation of esds in distances, angles and torsion angles; correlations between esds in cell parameters are only used when they are defined by crystal symmetry. An approximate (isotropic) treatment of cell esds is used for estimating esds involving l.s. planes.

### Fractional atomic coordinates and isotropic or equivalent isotropic displacement parameters (Å^2^)

**Table:**

	*x*	*y*	*z*	*U*_iso_*/*U*_eq_	Occ. (<1)
Ir1	0.47116 (2)	0.28881 (2)	0.65724 (2)	0.01084 (5)	
P1	0.48620 (10)	0.22720 (7)	0.81228 (7)	0.0129 (2)	
Cl2S	0.0421 (2)	0.01421 (16)	0.36372 (19)	0.0789 (7)	0.8
F3S	0.9219 (3)	0.5389 (2)	0.78303 (18)	0.0328 (6)	
F4S	0.8164 (2)	0.69052 (19)	0.76826 (18)	0.0289 (6)	
F1S	0.9941 (2)	0.6736 (2)	0.6995 (2)	0.0326 (6)	
F2S	0.8057 (2)	0.5665 (2)	0.63363 (17)	0.0320 (6)	
N1	0.2423 (3)	0.4168 (2)	0.6840 (2)	0.0140 (7)	
N3	0.4195 (3)	0.5201 (2)	0.7218 (2)	0.0143 (7)	
Cl1S	0.0513 (3)	−0.0240 (2)	0.1530 (2)	0.1174 (11)	0.8
N2	0.3241 (3)	0.5858 (3)	0.7354 (3)	0.0205 (7)	
C29	0.5370 (4)	0.3642 (3)	0.5344 (3)	0.0160 (8)	
H29	0.550441	0.445313	0.545582	0.019*	
C30	0.4086 (4)	0.3233 (3)	0.5020 (3)	0.0175 (8)	
H30	0.347227	0.380257	0.494070	0.021*	
C1	0.3731 (4)	0.4161 (3)	0.6917 (3)	0.0127 (8)	
C19	0.3798 (4)	0.2770 (3)	0.8936 (3)	0.0149 (8)	
C3	0.5541 (4)	0.5679 (3)	0.7448 (3)	0.0195 (9)	
H3A	0.568801	0.616238	0.694949	0.029*	
H3B	0.611578	0.510100	0.744217	0.029*	
H3C	0.572550	0.609368	0.810643	0.029*	
C26	0.6151 (4)	0.1796 (3)	0.6262 (3)	0.0192 (9)	
H26	0.655587	0.143814	0.684740	0.023*	
C18	0.7466 (4)	0.3189 (3)	0.8620 (3)	0.0165 (8)	
H18	0.735067	0.337400	0.795859	0.020*	
C25	0.4913 (4)	0.1322 (3)	0.5797 (3)	0.0175 (8)	
H25	0.459794	0.068703	0.611381	0.021*	
C20	0.3871 (4)	0.3897 (3)	0.9148 (3)	0.0157 (8)	
H20	0.444206	0.435293	0.886935	0.019*	
C13	0.6444 (4)	0.2631 (3)	0.8936 (3)	0.0143 (8)	
C28	0.6511 (4)	0.3133 (3)	0.5028 (3)	0.0216 (9)	
H28A	0.718341	0.371810	0.497250	0.026*	
H28B	0.621125	0.272044	0.436393	0.026*	
C4	0.1441 (4)	0.3222 (3)	0.6507 (3)	0.0177 (8)	
H4	0.190505	0.256841	0.639365	0.021*	
C2	0.2177 (4)	0.5198 (3)	0.7113 (3)	0.0201 (9)	
H2	0.133529	0.540672	0.712647	0.024*	
C7	0.4587 (4)	0.0800 (3)	0.8046 (3)	0.0172 (8)	
C17	0.8647 (4)	0.3474 (3)	0.9264 (3)	0.0207 (9)	
H17	0.933837	0.385545	0.904347	0.025*	
C31	0.3584 (4)	0.2175 (3)	0.4384 (3)	0.0213 (9)	
H31A	0.359934	0.227329	0.368231	0.026*	
H31B	0.267038	0.197832	0.443132	0.026*	
C24	0.2981 (4)	0.2109 (3)	0.9367 (3)	0.0188 (8)	
H24	0.293587	0.134215	0.923943	0.023*	
C16	0.8827 (4)	0.3207 (3)	1.0234 (3)	0.0245 (9)	
H16	0.964257	0.339914	1.067295	0.029*	
C12	0.5586 (5)	0.0158 (3)	0.8343 (3)	0.0244 (10)	
H12	0.642569	0.049271	0.865717	0.029*	
C14	0.6636 (4)	0.2380 (3)	0.9921 (3)	0.0186 (8)	
H14	0.594325	0.201175	1.015180	0.022*	
C23	0.2232 (4)	0.2573 (3)	0.9986 (3)	0.0227 (9)	
H23	0.167533	0.211953	1.027880	0.027*	
C32	0.4375 (4)	0.1250 (3)	0.4685 (3)	0.0216 (9)	
H32A	0.510754	0.124779	0.433141	0.026*	
H32B	0.382081	0.055348	0.447199	0.026*	
C8	0.3358 (5)	0.0294 (3)	0.7579 (3)	0.0250 (10)	
H8	0.267764	0.072071	0.735758	0.030*	
C5	0.0668 (4)	0.3029 (3)	0.7313 (3)	0.0208 (9)	
H5A	0.126737	0.297721	0.794004	0.031*	
H5B	0.009148	0.235171	0.712011	0.031*	
H5C	0.014971	0.363452	0.739793	0.031*	
C21	0.3119 (4)	0.4351 (3)	0.9760 (3)	0.0193 (9)	
H21	0.316717	0.511760	0.989634	0.023*	
C22	0.2290 (4)	0.3682 (3)	1.0178 (3)	0.0227 (9)	
H22	0.176535	0.399208	1.059377	0.027*	
C27	0.7118 (4)	0.2375 (4)	0.5753 (3)	0.0257 (10)	
H27A	0.753004	0.182627	0.538816	0.031*	
H27B	0.780945	0.280129	0.626494	0.031*	
C6	0.0561 (5)	0.3377 (4)	0.5533 (3)	0.0363 (12)	
H6A	0.005026	0.398302	0.563865	0.055*	
H6B	−0.002528	0.271299	0.528881	0.055*	
H6C	0.109113	0.353590	0.504214	0.055*	
C15	0.7816 (4)	0.2661 (4)	1.0557 (3)	0.0255 (10)	
H15	0.793659	0.247950	1.121984	0.031*	
C9	0.3128 (5)	−0.0837 (4)	0.7436 (3)	0.0345 (12)	
H9	0.229041	−0.118159	0.713045	0.041*	
C10	0.4134 (6)	−0.1451 (3)	0.7745 (3)	0.0350 (12)	
H10	0.397795	−0.222088	0.765248	0.042*	
B1S	0.8842 (5)	0.6174 (4)	0.7206 (3)	0.0215 (10)	
C11	0.5356 (5)	−0.0964 (4)	0.8182 (3)	0.0336 (12)	
H11	0.604132	−0.139631	0.837432	0.040*	
C1S	0.0793 (11)	0.0608 (8)	0.2588 (7)	0.096 (4)	0.8
H1SA	0.030619	0.124132	0.245361	0.115*	0.8
H1SB	0.172777	0.087820	0.273980	0.115*	0.8

### Atomic displacement parameters (Å^2^)

**Table:**

	*U*^11^	*U*^22^	*U*^33^	*U*^12^	*U*^13^	*U*^23^
Ir1	0.01151 (8)	0.01098 (7)	0.01084 (7)	0.00251 (5)	0.00350 (5)	0.00161 (5)
P1	0.0144 (5)	0.0126 (5)	0.0122 (5)	0.0019 (4)	0.0037 (4)	0.0022 (4)
Cl2S	0.0736 (16)	0.0489 (12)	0.0991 (17)	−0.0194 (10)	−0.0109 (13)	0.0124 (11)
F3S	0.0445 (18)	0.0312 (14)	0.0234 (13)	0.0127 (12)	0.0013 (12)	0.0094 (11)
F4S	0.0257 (15)	0.0321 (14)	0.0331 (14)	0.0090 (11)	0.0133 (12)	0.0044 (11)
F1S	0.0200 (14)	0.0391 (15)	0.0430 (16)	0.0048 (11)	0.0141 (12)	0.0089 (12)
F2S	0.0251 (15)	0.0475 (16)	0.0214 (13)	0.0043 (12)	−0.0001 (11)	0.0028 (11)
N1	0.0119 (18)	0.0140 (15)	0.0163 (16)	0.0019 (12)	0.0040 (13)	0.0001 (13)
N3	0.0144 (18)	0.0125 (15)	0.0179 (16)	0.0042 (12)	0.0058 (14)	0.0031 (13)
Cl1S	0.119 (3)	0.094 (2)	0.114 (2)	−0.0425 (18)	0.0074 (19)	−0.0362 (17)
N2	0.020 (2)	0.0168 (17)	0.0268 (19)	0.0063 (14)	0.0091 (16)	0.0023 (14)
C29	0.020 (2)	0.0178 (19)	0.0130 (18)	0.0026 (16)	0.0084 (17)	0.0074 (15)
C30	0.026 (2)	0.0182 (19)	0.0090 (18)	0.0066 (16)	0.0012 (16)	0.0050 (15)
C1	0.016 (2)	0.0139 (18)	0.0096 (17)	0.0004 (15)	0.0039 (15)	0.0061 (14)
C19	0.012 (2)	0.021 (2)	0.0114 (18)	0.0012 (15)	0.0016 (15)	0.0022 (15)
C3	0.018 (2)	0.0154 (19)	0.023 (2)	−0.0016 (16)	0.0024 (17)	0.0008 (16)
C26	0.023 (2)	0.020 (2)	0.019 (2)	0.0146 (17)	0.0085 (18)	0.0047 (16)
C18	0.018 (2)	0.021 (2)	0.0133 (18)	0.0054 (16)	0.0080 (16)	0.0031 (15)
C25	0.029 (2)	0.0067 (17)	0.0174 (19)	0.0075 (16)	0.0046 (18)	−0.0007 (15)
C20	0.014 (2)	0.020 (2)	0.0127 (18)	0.0001 (15)	0.0020 (16)	0.0019 (15)
C13	0.015 (2)	0.0149 (18)	0.0137 (18)	0.0066 (15)	0.0037 (16)	0.0005 (15)
C28	0.018 (2)	0.027 (2)	0.024 (2)	0.0031 (17)	0.0121 (18)	0.0066 (18)
C4	0.009 (2)	0.021 (2)	0.022 (2)	−0.0024 (15)	0.0046 (16)	−0.0028 (16)
C2	0.018 (2)	0.020 (2)	0.026 (2)	0.0087 (17)	0.0077 (18)	0.0046 (17)
C7	0.028 (2)	0.0140 (19)	0.0104 (18)	−0.0004 (16)	0.0078 (17)	0.0010 (15)
C17	0.011 (2)	0.030 (2)	0.021 (2)	0.0002 (17)	0.0063 (17)	0.0008 (17)
C31	0.025 (2)	0.021 (2)	0.015 (2)	−0.0013 (17)	−0.0001 (17)	−0.0019 (16)
C24	0.019 (2)	0.022 (2)	0.0157 (19)	0.0013 (16)	0.0036 (17)	0.0060 (16)
C16	0.016 (2)	0.037 (2)	0.019 (2)	0.0020 (18)	0.0031 (18)	−0.0009 (18)
C12	0.039 (3)	0.021 (2)	0.017 (2)	0.0062 (19)	0.0104 (19)	0.0039 (17)
C14	0.018 (2)	0.024 (2)	0.0142 (19)	0.0032 (16)	0.0027 (17)	0.0050 (16)
C23	0.017 (2)	0.034 (2)	0.017 (2)	−0.0045 (18)	0.0053 (17)	0.0049 (18)
C32	0.034 (3)	0.0152 (19)	0.0142 (19)	0.0006 (17)	0.0031 (18)	−0.0015 (16)
C8	0.036 (3)	0.020 (2)	0.019 (2)	−0.0045 (18)	0.0073 (19)	0.0023 (17)
C5	0.014 (2)	0.025 (2)	0.022 (2)	−0.0029 (16)	0.0009 (17)	0.0049 (17)
C21	0.016 (2)	0.024 (2)	0.0159 (19)	0.0030 (16)	0.0006 (17)	−0.0032 (16)
C22	0.017 (2)	0.035 (2)	0.017 (2)	0.0063 (18)	0.0062 (18)	0.0012 (18)
C27	0.022 (2)	0.036 (2)	0.024 (2)	0.0128 (19)	0.0104 (19)	0.0079 (19)
C6	0.021 (3)	0.065 (3)	0.021 (2)	−0.010 (2)	0.004 (2)	0.005 (2)
C15	0.027 (3)	0.037 (3)	0.014 (2)	0.0079 (19)	0.0050 (18)	0.0060 (18)
C9	0.054 (3)	0.026 (2)	0.022 (2)	−0.014 (2)	0.015 (2)	−0.0038 (19)
C10	0.069 (4)	0.015 (2)	0.026 (2)	0.001 (2)	0.023 (3)	0.0020 (18)
B1S	0.019 (3)	0.028 (3)	0.019 (2)	0.006 (2)	0.004 (2)	0.007 (2)
C11	0.059 (4)	0.025 (2)	0.025 (2)	0.017 (2)	0.018 (2)	0.0117 (19)
C1S	0.097 (8)	0.086 (7)	0.087 (7)	−0.048 (6)	0.018 (6)	−0.024 (6)

### Geometric parameters (Å, º)

**Table:**

Ir1—P1	2.3207 (12)	C28—C27	1.528 (6)
Ir1—C29	2.207 (4)	C4—H4	1.0000
Ir1—C30	2.211 (4)	C4—C5	1.524 (5)
Ir1—C1	2.034 (4)	C4—C6	1.520 (6)
Ir1—C26	2.198 (4)	C2—H2	0.9500
Ir1—C25	2.183 (3)	C7—C12	1.399 (6)
P1—C19	1.839 (4)	C7—C8	1.399 (6)
P1—C13	1.819 (4)	C17—H17	0.9500
P1—C7	1.820 (4)	C17—C16	1.391 (6)
Cl2S—C1S	1.709 (10)	C31—H31A	0.9900
F3S—B1S	1.395 (5)	C31—H31B	0.9900
F4S—B1S	1.398 (5)	C31—C32	1.522 (6)
F1S—B1S	1.395 (5)	C24—H24	0.9500
F2S—B1S	1.385 (5)	C24—C23	1.391 (5)
N1—C1	1.364 (5)	C16—H16	0.9500
N1—C4	1.477 (5)	C16—C15	1.382 (6)
N1—C2	1.362 (5)	C12—H12	0.9500
N3—N2	1.379 (4)	C12—C11	1.386 (6)
N3—C1	1.343 (5)	C14—H14	0.9500
N3—C3	1.456 (5)	C14—C15	1.376 (6)
Cl1S—C1S	1.683 (9)	C23—H23	0.9500
N2—C2	1.302 (5)	C23—C22	1.375 (6)
C29—H29	1.0000	C32—H32A	0.9900
C29—C30	1.380 (6)	C32—H32B	0.9900
C29—C28	1.524 (5)	C8—H8	0.9500
C30—H30	1.0000	C8—C9	1.396 (6)
C30—C31	1.508 (5)	C5—H5A	0.9800
C19—C20	1.398 (5)	C5—H5B	0.9800
C19—C24	1.391 (5)	C5—H5C	0.9800
C3—H3A	0.9800	C21—H21	0.9500
C3—H3B	0.9800	C21—C22	1.395 (6)
C3—H3C	0.9800	C22—H22	0.9500
C26—H26	1.0000	C27—H27A	0.9900
C26—C25	1.394 (6)	C27—H27B	0.9900
C26—C27	1.515 (6)	C6—H6A	0.9800
C18—H18	0.9500	C6—H6B	0.9800
C18—C13	1.393 (5)	C6—H6C	0.9800
C18—C17	1.383 (6)	C15—H15	0.9500
C25—H25	1.0000	C9—H9	0.9500
C25—C32	1.523 (5)	C9—C10	1.385 (7)
C20—H20	0.9500	C10—H10	0.9500
C20—C21	1.382 (5)	C10—C11	1.377 (7)
C13—C14	1.405 (5)	C11—H11	0.9500
C28—H28A	0.9900	C1S—H1SA	0.9900
C28—H28B	0.9900	C1S—H1SB	0.9900
			
C29—Ir1—P1	157.81 (11)	N2—C2—N1	111.5 (3)
C29—Ir1—C30	36.40 (15)	N2—C2—H2	124.3
C30—Ir1—P1	165.57 (11)	C12—C7—P1	122.4 (3)
C1—Ir1—P1	93.88 (10)	C12—C7—C8	119.1 (4)
C1—Ir1—C29	93.11 (14)	C8—C7—P1	118.1 (3)
C1—Ir1—C30	84.88 (14)	C18—C17—H17	119.8
C1—Ir1—C26	166.98 (15)	C18—C17—C16	120.4 (4)
C1—Ir1—C25	154.53 (15)	C16—C17—H17	119.8
C26—Ir1—P1	88.71 (11)	C30—C31—H31A	109.0
C26—Ir1—C29	80.05 (14)	C30—C31—H31B	109.0
C26—Ir1—C30	95.73 (15)	C30—C31—C32	112.8 (3)
C25—Ir1—P1	95.32 (10)	H31A—C31—H31B	107.8
C25—Ir1—C29	87.22 (14)	C32—C31—H31A	109.0
C25—Ir1—C30	80.35 (14)	C32—C31—H31B	109.0
C25—Ir1—C26	37.09 (15)	C19—C24—H24	120.0
C19—P1—Ir1	118.72 (13)	C23—C24—C19	119.9 (4)
C13—P1—Ir1	113.80 (12)	C23—C24—H24	120.0
C13—P1—C19	100.49 (17)	C17—C16—H16	120.1
C13—P1—C7	105.03 (18)	C15—C16—C17	119.7 (4)
C7—P1—Ir1	112.41 (12)	C15—C16—H16	120.1
C7—P1—C19	104.83 (17)	C7—C12—H12	119.9
C1—N1—C4	125.6 (3)	C11—C12—C7	120.3 (4)
C2—N1—C1	108.5 (3)	C11—C12—H12	119.9
C2—N1—C4	125.8 (3)	C13—C14—H14	119.6
N2—N3—C3	118.4 (3)	C15—C14—C13	120.7 (4)
C1—N3—N2	113.3 (3)	C15—C14—H14	119.6
C1—N3—C3	128.3 (3)	C24—C23—H23	119.7
C2—N2—N3	103.5 (3)	C22—C23—C24	120.6 (4)
Ir1—C29—H29	113.5	C22—C23—H23	119.7
C30—C29—Ir1	71.9 (2)	C25—C32—H32A	108.8
C30—C29—H29	113.5	C25—C32—H32B	108.8
C30—C29—C28	124.5 (4)	C31—C32—C25	113.6 (3)
C28—C29—Ir1	112.9 (3)	C31—C32—H32A	108.8
C28—C29—H29	113.5	C31—C32—H32B	108.8
Ir1—C30—H30	113.9	H32A—C32—H32B	107.7
C29—C30—Ir1	71.7 (2)	C7—C8—H8	119.9
C29—C30—H30	113.9	C9—C8—C7	120.2 (4)
C29—C30—C31	126.4 (4)	C9—C8—H8	119.9
C31—C30—Ir1	108.6 (2)	C4—C5—H5A	109.5
C31—C30—H30	113.9	C4—C5—H5B	109.5
N1—C1—Ir1	127.8 (3)	C4—C5—H5C	109.5
N3—C1—Ir1	128.8 (3)	H5A—C5—H5B	109.5
N3—C1—N1	103.3 (3)	H5A—C5—H5C	109.5
C20—C19—P1	116.0 (3)	H5B—C5—H5C	109.5
C24—C19—P1	124.7 (3)	C20—C21—H21	120.1
C24—C19—C20	119.3 (3)	C20—C21—C22	119.8 (4)
N3—C3—H3A	109.5	C22—C21—H21	120.1
N3—C3—H3B	109.5	C23—C22—C21	119.9 (4)
N3—C3—H3C	109.5	C23—C22—H22	120.1
H3A—C3—H3B	109.5	C21—C22—H22	120.1
H3A—C3—H3C	109.5	C26—C27—C28	113.5 (3)
H3B—C3—H3C	109.5	C26—C27—H27A	108.9
Ir1—C26—H26	114.2	C26—C27—H27B	108.9
C25—C26—Ir1	70.8 (2)	C28—C27—H27A	108.9
C25—C26—H26	114.2	C28—C27—H27B	108.9
C25—C26—C27	125.2 (4)	H27A—C27—H27B	107.7
C27—C26—Ir1	109.8 (3)	C4—C6—H6A	109.5
C27—C26—H26	114.2	C4—C6—H6B	109.5
C13—C18—H18	119.8	C4—C6—H6C	109.5
C17—C18—H18	119.8	H6A—C6—H6B	109.5
C17—C18—C13	120.3 (4)	H6A—C6—H6C	109.5
Ir1—C25—H25	113.6	H6B—C6—H6C	109.5
C26—C25—Ir1	72.1 (2)	C16—C15—H15	119.9
C26—C25—H25	113.6	C14—C15—C16	120.2 (4)
C26—C25—C32	124.4 (4)	C14—C15—H15	119.9
C32—C25—Ir1	112.4 (2)	C8—C9—H9	120.3
C32—C25—H25	113.6	C10—C9—C8	119.4 (5)
C19—C20—H20	119.8	C10—C9—H9	120.3
C21—C20—C19	120.4 (4)	C9—C10—H10	119.5
C21—C20—H20	119.8	C11—C10—C9	121.0 (4)
C18—C13—P1	121.7 (3)	C11—C10—H10	119.5
C18—C13—C14	118.6 (4)	F3S—B1S—F4S	109.3 (3)
C14—C13—P1	119.6 (3)	F3S—B1S—F1S	109.5 (4)
C29—C28—H28A	109.0	F1S—B1S—F4S	109.0 (4)
C29—C28—H28B	109.0	F2S—B1S—F3S	108.8 (4)
C29—C28—C27	113.0 (3)	F2S—B1S—F4S	110.3 (4)
H28A—C28—H28B	107.8	F2S—B1S—F1S	110.0 (3)
C27—C28—H28A	109.0	C12—C11—H11	120.0
C27—C28—H28B	109.0	C10—C11—C12	120.0 (4)
N1—C4—H4	108.2	C10—C11—H11	120.0
N1—C4—C5	110.0 (3)	Cl2S—C1S—H1SA	107.5
N1—C4—C6	110.6 (3)	Cl2S—C1S—H1SB	107.5
C5—C4—H4	108.2	Cl1S—C1S—Cl2S	119.2 (5)
C6—C4—H4	108.2	Cl1S—C1S—H1SA	107.5
C6—C4—C5	111.6 (3)	Cl1S—C1S—H1SB	107.5
N1—C2—H2	124.3	H1SA—C1S—H1SB	107.0
			
Ir1—P1—C19—C20	−56.0 (3)	C18—C13—C14—C15	−1.2 (6)
Ir1—P1—C19—C24	126.2 (3)	C18—C17—C16—C15	−0.6 (6)
Ir1—P1—C13—C18	−2.3 (3)	C25—C26—C27—C28	43.4 (5)
Ir1—P1—C13—C14	175.2 (3)	C20—C19—C24—C23	1.2 (6)
Ir1—P1—C7—C12	108.8 (3)	C20—C21—C22—C23	0.6 (6)
Ir1—P1—C7—C8	−64.6 (3)	C13—P1—C19—C20	68.7 (3)
Ir1—C29—C30—C31	−99.9 (4)	C13—P1—C19—C24	−109.1 (4)
Ir1—C29—C28—C27	−11.3 (4)	C13—P1—C7—C12	−15.4 (4)
Ir1—C30—C31—C32	−38.6 (4)	C13—P1—C7—C8	171.2 (3)
Ir1—C26—C25—C32	105.3 (3)	C13—C18—C17—C16	0.1 (6)
Ir1—C26—C27—C28	−36.6 (4)	C13—C14—C15—C16	0.7 (6)
Ir1—C25—C32—C31	−11.4 (4)	C28—C29—C30—Ir1	105.8 (4)
P1—C19—C20—C21	−179.4 (3)	C28—C29—C30—C31	5.9 (6)
P1—C19—C24—C23	178.9 (3)	C4—N1—C1—Ir1	1.7 (5)
P1—C13—C14—C15	−178.8 (3)	C4—N1—C1—N3	178.4 (3)
P1—C7—C12—C11	−173.8 (3)	C4—N1—C2—N2	−178.9 (3)
P1—C7—C8—C9	175.3 (3)	C2—N1—C1—Ir1	−177.7 (3)
N3—N2—C2—N1	0.2 (4)	C2—N1—C1—N3	−1.1 (4)
N2—N3—C1—Ir1	177.9 (3)	C2—N1—C4—C5	−58.9 (5)
N2—N3—C1—N1	1.2 (4)	C2—N1—C4—C6	64.9 (5)
C29—C30—C31—C32	41.9 (5)	C7—P1—C19—C20	177.5 (3)
C29—C28—C27—C26	31.9 (5)	C7—P1—C19—C24	−0.3 (4)
C30—C29—C28—C27	−94.5 (5)	C7—P1—C13—C18	121.0 (3)
C30—C31—C32—C25	33.8 (5)	C7—P1—C13—C14	−61.5 (3)
C1—N1—C4—C5	121.8 (4)	C7—C12—C11—C10	−1.2 (6)
C1—N1—C4—C6	−114.5 (4)	C7—C8—C9—C10	−1.2 (6)
C1—N1—C2—N2	0.5 (4)	C17—C18—C13—P1	178.3 (3)
C1—N3—N2—C2	−0.9 (4)	C17—C18—C13—C14	0.8 (5)
C19—P1—C13—C18	−130.4 (3)	C17—C16—C15—C14	0.2 (6)
C19—P1—C13—C14	47.2 (3)	C24—C19—C20—C21	−1.5 (6)
C19—P1—C7—C12	−120.9 (3)	C24—C23—C22—C21	−0.9 (6)
C19—P1—C7—C8	65.7 (3)	C12—C7—C8—C9	1.6 (6)
C19—C20—C21—C22	0.6 (6)	C8—C7—C12—C11	−0.4 (6)
C19—C24—C23—C22	0.0 (6)	C8—C9—C10—C11	−0.4 (6)
C3—N3—N2—C2	−178.4 (3)	C27—C26—C25—Ir1	−101.1 (4)
C3—N3—C1—Ir1	−4.9 (5)	C27—C26—C25—C32	4.2 (6)
C3—N3—C1—N1	178.4 (3)	C9—C10—C11—C12	1.6 (6)
C26—C25—C32—C31	−94.5 (5)		

### Hydrogen-bond geometry (Å, º)

**Table:**

*D*—H···*A*	*D*—H	H···*A*	*D*···*A*	*D*—H···*A*
C2—H2···F3*S*^i^	0.95	2.60	3.471 (5)	153
C2—H2···F1*S*^i^	0.95	2.30	3.154 (5)	149
C5—H5*C*···F3*S*^i^	0.98	2.54	3.505 (5)	169
C6—H6*C*···F2*S*^ii^	0.98	2.50	3.451 (5)	163
C10—H10···N2^iii^	0.95	2.42	3.364 (6)	172

Symmetry codes: (i) *x*−1, *y*, *z*; (ii) −*x*+1, −*y*+1, −*z*+1; (iii) *x*, *y*−1, *z*.
